# Computed Tomography versus Sleep Endoscopy (DISE) to Predict the Effectiveness of Mandibular Advancement Devices in Adult Patients with Obstructive Sleep Apnea: A Protocol for Systematic Review

**DOI:** 10.3390/jcm12196328

**Published:** 2023-10-01

**Authors:** Pedro Cebola, Cristina Caroça, Helena Donato, Ana Campos, Sara Simões Dias, João Paço, Cristina Manso

**Affiliations:** 1 Egas Moniz Center for Interdisciplinary Research (CiiEM), Egas Moniz School of Health & Science, 2829-511 Almada, Portugal; pedro.cebola@cuf.pt (P.C.); cmanso@egasmoniz.edu.pt (C.M.); 2 CUF Tejo Hospital, 1300-352 Lisboa, Portugal; ana.t.campos@cuf.pt (A.C.); joao.paco@cuf.pt (J.P.); 3 CHRC, NOVA Medical School, Universidade Nova de Lisboa (NMS/UNL), 1169-056 Lisboa, Portugal; 4 Documentation and Scientific Information Service, Centro Hospitalar e Universitário de Coimbra, 3000-075 Coimbra, Portugal; helenadonato@chuc.min-saude.pt; 5 EpiDoC Unit, Centro de Estudos de Doenças Crónicas (CEDOC) da NOVA Medical School, Universidade Nova de Lisboa (NMS/UNL), CEDOC—Campus Sant’Ana, Pólo de Investigação, NMS, UNL, Edifício Amarelo, Rua do Instituto Bacteriológico no. 5, 1150-082 Lisboa, Portugal; sara.dias@nms.unl.pt; 6 EpiSaúde Sociedade Científica, 7005-837 Évora, Portugal; 7 Escola Superior de Saúde do Instituto Politécnico de Leiria, Unidade de Investigação em Saúde (UI), 2411-901 Leiria, Portugal

**Keywords:** sleep apnea, oral appliance, computed tomography, sleep endoscopy

## Abstract

Obstructive sleep apnea is a sleep disorder with a high prevalence in the world population. The mandibular advancement device is one of the options for treating obstructive sleep apnea. Neck computed tomography and drug-induced sleep endoscopy are complementary diagnostic tests that may help predict the effectiveness of mandibular advancement devices. This study aims to analyze the best method for predicting the effectiveness of mandibular advancement devices in the therapeutic approach to obstructive sleep apnea. PubMed, Embase, Cochrane Central Register of Controlled Trials (CENTRAL), and Web of Science Core Collection databases will be comprehensively searched. We will include randomized clinical trials, non-randomized prospective or retrospective clinical studies, case controls, cohort studies, and case series. Two authors will independently conduct data extraction and assess the literature quality of the studies. The analysis of the included literature will be conducted by Revman 5.3 software. The outcomes that will be analyzed are craniofacial characteristics, cephalometric assessments, site and type of obstruction of the upper airway, mean values of the apnea–hypopnea index, and SaO_2_ verified in the initial and follow-up polysomnography. This study will provide reliable, evidence-based support for the clinical application of mandibular advancement devices for obstructive sleep apnea.

## 1. Introduction

Obstructive sleep apnea syndrome (OSA) is a sleep disorder that has a high prevalence in the world population; it is characterized by a partial or total collapse of the upper airways (UAs) during sleep due to recurrent episodes of apnea or hypopnea [1]. The recovery of regular breathing after this type of episode occurs with respiratory effort, leading to sleep fragmentation, decreased sleep quality, and daytime sleepiness [2]. 

The epidemiology of people affected by OSA varies between 14% and 84% in men and from 5% to 61% in women. However, it is thought that the number is undervalued and that many more people with OSA do not have a definitive diagnosis [2,3].

According to international recommendations, the diagnosis of OSA is performed through a sleep examination, namely a polysomnography (PSG) or home sleep apnea testing (HSAT) [4]. Apnea is defined as a 90% reduction in airflow lasting at least 10 s. Hypopnea is defined as a decrease in airflow of at least 50% and a decrease in oxygen saturation of 3% for at least 10 s. However, accredited sleep centers are allowed to classify hypopneas when there is an oxygen desaturation ≥4% of pre-event baseline, in adults. The apnea–hypopnea index (AHI), which corresponds to the ratio of apnea/hypopnea per hour of sleep, defines the severity of OSA. When AHI is under 5, it is defined as if there is no OSA; when the AHI is between 5 and 15, it is considered to be mild OSA; an AHI between 15 and 30 is considered to be moderate OSA; and when this index is above 30, it is considered to be severe OSA. Sleep apnea events identified in PSG in asymptomatic individuals are only considered to be OSA when the AHI is above 15 [5].

OSA is generally associated with important comorbidities, such as cardiovascular and metabolic pathologies, which are observed in 50% of patients with OSA. One of the most prevalent examples is persistent secondary arterial hypertension and nocturnal profiles of non-dipping hypertension. The prevalence of OSA in patients with type 1 diabetes mellitus is also high [6]. It is also associated with cerebrovascular diseases, such as cerebral infarction, transient ischemic attack, and ischemic or hemorrhagic cerebrovascular accidents, with a combined prevalence of 62% in moderate OSA, which is characterized by an apnea–hypopnea index (AHI) of > 15/h, and 30% in severe OSA with an AHI of >30/h [7].

Since the first publication of the treatment of OSA with continuous positive airway pressure (CPAP) in 1981, the therapy has been considered the gold standard for symptomatic OSA, and OSA patients with comorbidities or with moderate to severe disease diagnosed via polysomnography (PSG) [1].

This CPAP therapy is also the gold standard in AHI reduction, as it improves the patient’s quality of life, reduces the risk of motor vehicle accidents, and reduces cardiovascular events and mortality associated with OSA. However, between 30% and 50% of patients prescribed CPAP fail to maintain long-term adherence [8,9].

Given not everyone will be successful with CPAP, alternative treatments are needed. Mandibular advancement devices (MADs) were the first non-invasive alternative treatments for OSA. The MAD serves the purpose of positioning the mandible forward to manage the openness of the upper airway effectively and, thus, to avoid a limitation of the airflow during sleep, also allowing a total or partial reduction in the AHI. A therapeutic approach with MADs may not be as effective as that with CPAP in controlling UA obstruction, but there is a known superior clinical and scientific adherence to this therapy [9]. Despite the scientific documentation regarding the lower effectiveness of MADs compared to CPAP, there has been an increase in the publication of articles suggesting that the therapeutic approach to OSA with MADs has proven to be effective in improving the parameters of PSG indices, objective and subjective measures of sleepiness, blood pressure, neuropsychological functioning, and quality of life. It is suggested that MADs can cause a reduction of ≥50% in the AHI in approximately 60–70% of patients, and that in about 35–40% of patients, the AHI is <5 for events h−1 [10].

The two complementary diagnostic tests (CT and DISE) might not be commonly employed for obstructive sleep apnea, and they can incur a substantial cost [11].

MADs can be standardized (performed without impressions of the dental arches) or, customized (in which there is an impression of the dental arches), and can even be titratable or non-titratable. The non-titratable MAD keeps the mandible in a unique position of mandibular protrusion, without the possibility of changing it during treatment. On the other hand, the titratable MAD presents a mechanism that allows a modification of the variable amounts of mandibular protrusion depending on the patient’s therapeutic response. Increasing jaw protrusion is considered analogous to CPAP titration [12].

Anatomical and neuromuscular factors play an essential role in the pathophysiology of OSA. Diagnostic imaging methods may play a useful role in the evaluation of OSA to predict which patients would respond to MADs [13]. One of them is computed tomography (CT), which allows detailed images of the bone and soft tissues of the upper airways, from the nasopharynx to the larynx, to be taken. After acquiring high-resolution images with a 1 to 2 mm thickness in the axial plane, length and cross-section measurements are performed with high precision [14]. 

Despite being a static and two-dimensional assessment of the three-dimensional dynamic anatomical structures of the head and neck, CT is considered to be a useful predictive tool, with significant differences in these measurements being verified between asymptomatic patients and patients with OSA. In addition to conventional vertical cephalometry, the technique of cephalometry in the supine position was introduced in order to verify the effect of changing body position on the anatomy and function of the upper airways in patients with OSA. With this analysis, it was observed that there are morphological predictors that negatively affect the possibility of OSA, such as retrognathia, micrognathia, long face, an inferior positioning of the hyoid bone, an accentuated mandibular plane, a narrowing of the upper airways, a soft palate, and a large tongue. This cephalometric analysis can be performed through CT and is not used exclusively for diagnostic purposes, but also to assess bones and soft tissues in the preoperative period in patients scheduled to undergo surgical therapy for OSA [15]. 

Several types of surgical procedures are described as well as the important role of positional therapy that has been introduced over the years. These therapies can be used alone or in combination, always with the aim of obtaining the best adherence to therapy and treatment [16]. It is thus clear that personalized treatment is essential to reconciling good results with long-term adherence. With this objective, several tools were introduced to assist in the therapeutic decision, among them drug-induced sleep endoscopy (DISE). This procedure, introduced in 1991, allows a dynamic assessment of flutter and collapse sites using a flexible nasopharyngoscopy to visualize the UA under sedation [17].

The literature suggests that around 50% of surgical treatment plans are changed after performing DISE, when compared to other assessments in which the patient is awake. It is also suggested that certain aspects observed during DISE are associated with a better or worse outcome of the selected therapeutic approach [16].

Of course, DISE does not only offer indications or advantages. Contraindications are associated with the anesthetic risk profile. Absolute contractions include the following: ASA 4, pregnancy, and allergy to DISE sedative agents. Morbid obesity is considered a relative contraindication, as these patients generally have no indication and are not good candidates for UA or MAD surgery. However, the morbidly obese are not absolutely excluded for DISE when the patient has specific features in the UA that can be corrected or improved, which have to be carefully analyzed when considering surgical treatments or the MAD [16].

In order to optimize the prediction of therapeutic success with MADs, intraoral devices (titratable) and mandibular advancement maneuvers (chin lift and jaw thrust (Esmarch)) can be applied during DISE, which may be undesirable due to disturbing stimuli, which are potentially associated with micro-arousals of the patient [17].

This systematic review aims to analyze which is the best method for predicting the effectiveness of a MAD in the therapeutic approach to OSA, which is an important topic since there is a demand for us to be able to increasingly individualize the treatment for each patient and terms security in prescribing MAD treatment.

## 2. Materials and Methods

### 2.1. Protocol Register and Ethics

This systematic review protocol has been drafted under the guidance of the Preferred Reporting Items for Systematic Reviews and Meta-Analysis Protocols (PRISMA-P) [18]. Moreover, this protocol was registered in the PROSPERO database on 2 November 2021 (registration number: CRD42021282845).

Since this is a protocol with no patient recruitment and personal information collection, approval by the ethics committee is not required.

### 2.2. Research Question

What will be the most effective complementary diagnostic method in terms of Computed tomography (CT) with cephalometry versus drug-induced sleep endoscopy (DISE) with the use of propofol as a sedative agent and the use of the VOTE system to evaluate upper airway (UA) obstructions and predict a favorable outcome in treatment with a mandibular advancement device (MAD) in adult patients with mild to moderate (AHI between 5 and 30/h) obstructive sleep apnea (OSA)?

#### Review Question

Based on the PICO approach, the framework for the research question is as follows:

Patient: Adult human patients of any ethnicity/sex who are proposed a therapeutic approach to OSA with MADs;

Intervention: OSA patients who underwent DISE or computed tomography (CT) with cephalometry to evaluate upper airway (UA) obstructions;

Comparison: OSA patients who performed DISE or CT with cephalometry to evaluate UA obstructions and predict MAD results; 

Outcome: The upper airway obstructions observed on CT with cephalometry predict/influence the outcome of MAD treatment more effectively than they do when using DISE.

### 2.3. Eligibility Criteria

#### 2.3.1. Types of Studies

We will include randomized clinical trials, non-randomized prospective or retrospective clinical studies, case controls, cohort studies, and case series. Due to the limited quantity of existing studies on the subject, we chose to expand the scope of our research.

#### 2.3.2. Patients

The participants in our study will consist of adults aged between 18 and 65 years old who have been diagnosed with mild or moderate OSA (indicated by an AHI between 5 and 30/h) using PSG and HSAT based on established diagnostic criteria. There will be no gender or ethnicity restrictions.

#### 2.3.3. Intervention

Patients in the treatment group DISE were treated with the use of propofol as a sedative agent and the use of the VOTE system to classify UA obstruction to predict MAD treatment, while patients in the control group underwent neck CT with every kind of cephalometry to predict MAD treatment.

#### 2.3.4. Outcome Indicators

(1) Main outcomes: The primary measurement outcomes will be craniofacial characteristics, cephalometric assessments, and the site and type of obstruction of the UA. 

(2) Additional outcomes: These outcomes will also be important for our work. These will include the following: mean values of AHI (AHI changes such as a >50% improvement and improvement to <15/h or <5/h), average SaO_2_, time under 90% SaO_2_ (T90), and improvement in the Epworth sleepiness scale (ESS) and/or in the Pittsburgh Sleep Quality Index (PSQI) verified in the initial and follow-up PSG; heart rate, SaO_2_, and bispectral index score (BIS) were revealed during DISE.

### 2.4. Exclusion Criteria 

The exclusion criteria that were defined by our working group include the following: (1) studies that do not present a clear and reproducible methodology; (2) studies with child and adolescent patients, and patients with craniofacial malformations; (3) animal studies, conference abstracts, editorials, case reports, book chapters, and review articles.

### 2.5. Information Sources 

The search will use a sensitive subject and topic-based strategy. We will search the following databases: PubMed, Embase, Cochrane Central Register of Controlled Trials (CENTRAL), and Web of Science Core Collection. Hand searching will also be performed from the reference/citation lists of the full-text articles that are eligible for inclusion in this systematic review.

### 2.6. Search Strategy 

We will systematically search the following databases: PubMed, Embase, Cochrane Central Register of Controlled Trials (CENTRAL), and Web of Science. Hand searching will also be performed from the reference/citation lists of the full-text articles that are eligible for inclusion in this systematic review.

The main search terms will be as follows:

- “obstructive sleep apnea”, “obstructive sleep apnea syndrome”, “sleep apnea syndrome”, “snoring”, ”sleep-related breathing disorder*”, ”sleep respiratory disorder*”, “sleep-disordered breathing”, and “OSA”;
- “Prediction”—“predict*”, “prognostic*”;
- “anatomy-base outcome” “anatomic obstruction”, “computed tomography”, “CT”, “Drug-induced sleep endoscopy”, “DISE”, “sleep endoscopy”, “cephalometry”, and “cephalometric”;
- “Oral appliances”—“mandibular advancement device”, “mandibular advancement appliance”, “mandibular advancement splint”, “mandibular repositioning device”, “mandibular repositioning appliance”, “mandibular repositioning splint”, “oral appliance”, “oral device”, “dental appliance”, and “dental device”.

Boolean operators (AND/OR) will be applied to combine searches. Only studies published after 1990 will be included, and these will be written in English since DISE only began to be carried out in 1991 [17]; the reason it will be written in English is because this will help ensure that the review includes a representative sample of the most widely recognized studies in the scientific community.

### 2.7. Data Filtering and Extraction

#### Eligible Studies Will Be Selected in Two Phases

During the first phase, the title and abstract of the studies will be reviewed by two reviewers. Inclusion criteria include the following: (1) adults diagnosed with mild to moderate OSA (indicated by an AHI between 5 and 30/h) via polysomnography (PSG) recordings and treated using MADs who underwent CT with cephalometry or DISE with the use of propofol as a sedative agent and the use of the VOTE system to classify UA obstruction, before using MADs; (2) treatment outcomes assessed via a second PSG recording; (3) the evaluation of UA obstructions using CT with cephalometry and/or DISE on OSA patients with MAD treatment or assessments of the predictors of MAD treatment outcomes in OSA patients; (4) studies published after 1990 and written in English. Exclusion criteria include the following: (1) no treatment modalities mentioned or a therapy other than that using MADs; (2) patients with an absence of OSA (AHI < 5/h) or severe OSA (AHI > 30) patients; (3) editorials, reviews, conference abstracts, case reports, and book chapters; (4) patients under 18 years of age and over 65 years of age; (5) a lack of a clear description of the inclusion and exclusion criteria in the studies; (6) studies that do not present a clear and reproducible methodology.

During the second phase, the full texts of all potentially eligible studies identified during the first phase will be reviewed independently by two reviewers. During the full-text assessment, irrelevant studies will be excluded based on the same inclusion and exclusion criteria mentioned above. During the review process, discrepancies between the two reviewers will be solved via a discussion with another reviewer to reach a consensus.

Information will be extracted from the selected studies by one reviewer and confirmed by the other reviewer. 

We will provide a narrative synthesis of the findings from the included studies. 

The analysis will be conducted using Reviewer Manager Version 5.3. and extracted studies will be classified according to each target outcome.

A manual search of potentially missing studies will be completed via screening the references of studies identified in the second phase. 

**Figure 1:**
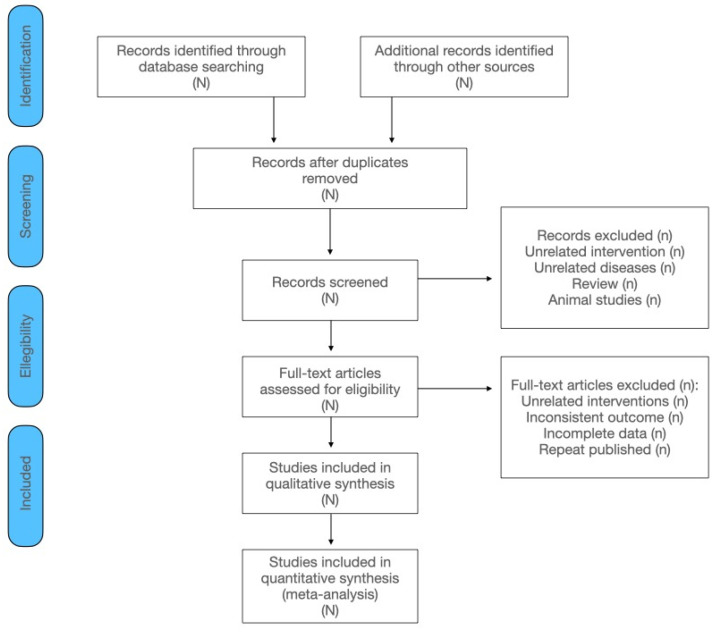
The process of literature filtering.

The process of literature filtering is shown in Figure 1.

### 2.8. Literature Quality (Bias) Assessment

The methodological quality of the studies will be assessed and scored to evaluate the risk of bias and applicability of primary diagnostic accuracy studies (QUADAS-2). This will consist of four key domains: patient selection, an index test, reference standard, and flow and timing.

Each is assessed in terms of risk of bias, and the first three are assessed in terms of concerns regarding applicability. Signaling questions are included to assist in judgments about the risk of bias. QUADAS-2 will be applied in four phases: 1—summarize the review question; 2—tailor the tool to the review and produce review-specific guidance; 3—construct a flow diagram for the primary study; 4—assess the risk of bias and concerns regarding applicability.

### 2.9. Statistical Analysis

We will apply RevMan 5.4 software for statistical analysis in this study. Risk ratios and 95% confidence intervals (CIs) will be obtained for enumeration data, while mean difference or standardized mean difference and 95% CIs will be used to calculate continuous outcome data. The heterogeneity between the attempts will be identified via the statistics of the I^2^ and Chi-squared tests. If we observe high heterogeneity in the included studies (I^2^ > 50%), we will use a random effects model to group the data among the studies.

## 3. Discussion

There is no consensus on the criteria for the success of MAD therapy; however, the most recent scientific evidence suggests that a low body mass index (BMI), supine-dominant OSA, neck circumference with a diameter of less than 40 cm, and gender may influence the success rate of treatment with MADs [17].

The effectiveness of the MAD in patients with OSA may vary depending on the severity of the condition. Patients with severe OSA are less likely to experience success with MADs compared to those with mild or moderate OSA. Factors such as large palatine tonsils or pronounced pharyngeal pillars can lead to poorer outcomes with MADs due to the partial collapse or compression of the base of the tongue. With this background knowledge, it becomes evident that pretreatment measures targeting an anteriorly located soft palate or base of the tongue could potentially improve the results of MAD treatment [19].

Recent technological advances in CT have also allowed volume measurements via the construction of three-dimensional images of the skull, facial structures, mandible, hyoid bone, spine, and airways with specific software, such as Sicat air^®^ [15]. Another potential predictor of MAD success, as analyzed through CT, is the angle of the skull base and the distance between the sella turcica and the deepest point in the posterior cranial fossa [20].

While CT is valuable for specific cases, it is not recommended for routine use due to its associated costs, limited accessibility, and relatively high radiation doses [10,21].

A variable was added to DISE to assess and plan the best therapeutic approach for OSA [22,23]. This variable is personalized bite registration, carried out in a consultation before the DISE by a sleep dentist. It represents the maximum comfortable protrusive position (MCP) of the patient. Thus, the prognostic value of the DISE procedure with bite registration in MCP is evaluated relative to the result of the treatment with MADs [24]. 

The literature suggests that patients who show a positive response to MAD treatment often experience a significant increase in the total UA volume, highlighting that the effectiveness of MADs is associated with a greater increase in UA volume. Conversely, the absence of an increase in velopharyngeal volume seems to be linked to the deterioration of the UA [13,25]. Moreover, there is evidence suggesting an association between the response to MAD treatment and the total volume of the upper airways, particularly in the velopharyngeal region [13,26,27].

The personalization of approaches to diagnosing and treating OSA is crucial for optimizing patient outcomes. Personalized medicine in OSA represents a new strategic approach, as traditional methods have not effectively addressed all of the critical issues in the diagnosis and therapeutic management of OSA. A personalized therapeutic approach aims to adequately address the specific requirements of each individual patient, thereby achieving appropriate and optimal treatment. Despite the advancements in diagnosis and treatment over recent decades, adopting a personalized and comprehensive approach will lead to better and more effective management for patients with OSA [28].

Both neck CT and DISE enable a three-dimensional assessment of the upper airway. While neck CT is performed with the patient awake and DISE involves pharmacologically induced sleep, there is some overlap between the two techniques. CT data can provide insights into obstructions related to the lateral walls of the oropharynx, as highlighted by Zhang et al. in 2014. Consequently, there is a suggestion that a partial substitution of DISE, which requires a trained team and a specially equipped room to be available, can be time-consuming, expensive, and requires pharmacological sleep induction [28].

There are relatively few studies that directly compare DISE with neck CT, and although these two exams are very useful for evaluating patients with OSA, each one of them gives some different information [29], such as those already discussed above. The first gives dynamic information and the second provides more static information [16]. However, UA morphology, which is shown and analyzed via DISE and/or neck CT, does not fully explain the inherent complexity of the pathophysiology of OSA. This condition is multifactorial and cannot be explained solely via observing the anatomical pathway. The arousal threshold, respiratory control stability, and genioglossus muscle responsiveness are examples of non-anatomical variables that are of great importance in OSA, must be taken into account, and can vary significantly in a sleeping patient compared to an awake one [30].

There is scientific evidence indicating that a potentially less intrusive approach for evaluating the prediction of treatment success and identifying patients who will or will not respond to a MAD is the use of standard or provisional MADs. These options tend to be more affordable and frequently demonstrate effectiveness in treating OSA [31]. 

Our proposed systematic review will be reported in accordance with the guidelines provided in the Preferred Reporting Items for Systematic Reviews and Meta-Analysis (PRISMA) statement [18]. 

An important step towards increasing the transparency of the research carried out and the reliability of public articles is this process of reporting and publishing protocols. Some journals, for example, suggest that the peer review process should include analysis of the randomized trial protocol. From 1 March 2014 to 8 June 2014, 66 of BioMed Central’s 258 open access journals published 4158 trial protocols, including 1026 in the journal Trials. The Biomed Central journal called Systematic Reviews is committed to publishing systematic review products [32] and since it was launched in February 2012 until 8 June 2014 it has published 142 systematic review protocols [18].

In the “instructions to authors” of systematic review proposals for various journals, funding agencies and systematic review organizations are encouraged to endorse PRISMA-P 2015. In guidance for publication applicants and its use during the peer review process as well its implementation, it is considered advantageous. The PRISMA-P checklist and its “explanation and elaboration” document are suggested to journal reviewers [29] in order to guide them. In protocol documentation, the integrity of reports from systematic review protocols will be superior, strengthening the methodological quality and reliability of systematic reviews when they are completed [18].

This systematic review will present the current evidence related to these two complementary diagnostic methods, neck CT and DISE, in the effective prediction of MADs for the therapeutic approach of OSA, through an exhaustive, transparent, and reproducible systematic literature search. In this systematic review protocol, we describe the multiple inclusion and exclusion criteria of eligible studies, which cover the target population, context, study design, intervention and comparison, and outcome measures. We determined the research strategy and databases for the search, followed by an extraction strategy and one for data synthesis. This process will be carried out explicitly and conducted in such a way as to minimize the risk of errors and biases. Through the use of adapted and standardized tools, the included studies will be submitted for a critical evaluation of the outcomes.

Some potential limitations that we anticipate from this review include that there must be some heterogeneity in the methods of evaluating the studies and there are few randomized clinical trials on the subject. These limitations may influence the ability to aggregate, transfer, and generalize the extracted data. Another possible limitation of our systematic review is the information bias that may exist due to our restriction to studies reported in the English language and in adult populations. However, this systematic review is opportune since there is a dissemination in the use of MADs, and we will approach and identify gaps and benefits of the use of neck CT and DISE for the possibility of predicting success in the therapy for OSA, as it is important to have several tools at our disposal for the benefit of an evidence-based individualized therapeutic approach.

## 4. Conclusions

We believe that this comprehensive systematic review has the potential to yield numerous valuable recommendations for patients and researchers alike. These recommendations could serve as essential guidance in determining the most effective therapy for specific patient cases and also inform the design of future studies in this field.

This systematic review’s potential to offer actionable recommendations and shape future research underscores its significance as a valuable resource in the medical sleep community. The knowledge acquired from this study has the power to positively impact patient care, inspire innovative research, and drive continuous improvement in healthcare practices.

## Data Availability

Not applicable.
